# Thoracodorsal Artery Perforator Flap Versus Split-Thickness Skin Graft Reconstruction for Advanced Axillary Hidradenitis Suppurativa: Long-Term Outcomes

**DOI:** 10.3390/jcm15114395

**Published:** 2026-06-05

**Authors:** Süleyman Çeçen, Menekşe Kastamoni Başkan, Güzin Yeşim Özgenel, Selçuk Akın

**Affiliations:** Plastic Reconstructive and Aesthetic Surgery Department, Faculty of Medicine, Bursa Uludağ University, Bursa 16059, Türkiye; mkastamoni@uludag.edu.tr (M.K.B.); yesimozgenel@uludag.edu.tr (G.Y.Ö.); sakin@uludag.edu.tr (S.A.)

**Keywords:** hidradenitis suppurativa, thoracodorsal artery perforator flap, split-thickness skin graft, axillary reconstruction, quality of life

## Abstract

**Background**: Axillary hidradenitis suppurativa (HS) often requires wide surgical excision and reconstruction. Thoracodorsal artery perforator (TDAP) flaps and split-thickness skin grafts (STSGs) are common options, but comparative long-term data are insufficient. **Methods**: In this single-center retrospective study, patients aged ≥ 17 years with Hurley stage II–III axillary HS underwent wide excision followed by TDAP flap or STSG reconstruction. Demographic variables, surgical characteristics, complications, recurrence, shoulder mobility, and dermatology-specific quality-of-life outcomes assessed using the Dermatology Life Quality Index (DLQI) were analyzed. **Results**: In total, 35 reconstructions were reviewed: TDAP (n = 15, 42.9%) and STSG (n = 20, 57.1%). Follow-up was longer for TDAP (28.53 ± 16.38 vs. 19.65 ± 28.06 months; *p* = 0.014). Mean defect size was 105.47 ± 26.29 cm^2^ (TDAP) vs. 164.65 ± 77.99 cm^2^ (STSG; *p* = 0.116). Both groups showed significant improvement in DLQI from preoperative to postoperative assessments (TDAP: +20.87; Graft: +18.50; both *p* < 0.0001), with no significant postoperative difference (*p* = 0.9608). Smokers had higher preoperative DLQI scores than non-smokers (+5.72; *p* = 0.0051), but postoperative outcomes were similar (*p* = 0.5908). **Conclusions**: Both reconstructions after wide axillary excision provided durable coverage, low complication rates, and significant improvement in quality of life. Incorporating patient-reported and functional outcomes into reconstructive planning may optimize surgical decision-making for axillary HS.

## 1. Introduction

Hidradenitis suppurativa (HS) is a chronic, recurrent, and progressive inflammatory skin disorder that can markedly impair patients’ quality of life, particularly when it involves functionally critical anatomical regions such as the axillae [1,2]. While early-stage disease may respond to medical therapy, wide surgical excision remains the most effective treatment approach in advanced cases (Hurley stage II–III) [3]. Reconstruction of the resulting soft-tissue defects following surgical excision is a multidisciplinary process that aims not only to achieve wound closure, but also to preserve function, improve aesthetic appearance, and reduce the risk of recurrence [4].

In the axilla—a highly mobile, moist, and perspiration-prone anatomical area—the choice of reconstruction method is a critical determinant of treatment success. STSG is frequently selected due to its technical simplicity, low cost, and suitability for covering large defects [5]. However, they have notable drawbacks, including susceptibility to environmental influences, prolonged healing time, risk of hypertrophic scarring, and secondary contracture. In high-friction and moisture-rich areas such as the axilla, graft-based reconstructions also carry an increased risk of recurrence. In the axillary region, exposure to moisture, friction, and shear forces may compromise graft adherence and contribute to delayed healing or hypertrophic scarring, particularly following graft-based reconstruction.

As an alternative, perforator-based flaps—such as the TDAP flap—are theoretically associated with advantages, including robust vascularity, durable tissue coverage, and potentially reduced risk of secondary contracture, as suggested by the existing literature [6,7]. Nevertheless, these techniques require greater surgical expertise and are associated with longer operative times and potential donor-site morbidity.

This study aims to perform a comprehensive, long-term comparison of outcomes between TDAP flap reconstruction and STSG in patients undergoing wide surgical excision for advanced (Hurley stage II–III) axillary hidradenitis suppurativa. The goal is to contribute to the determination of the optimal surgical approach by evaluating the extended outcomes of these two commonly employed reconstructive techniques.

## 2. Materials and Methods

Between January 2017 and December 2024, a total of 65 patients underwent surgical treatment for axillary hidradenitis suppurativa at our institution. Among these, patients treated with primary closure, secondary intention healing, or with insufficient follow-up were excluded. The final study cohort comprised patients who underwent wide excision followed by reconstruction with either a TDAP flap or STSG and had a minimum follow-up of six months.

This single-center, retrospective, comparative clinical study included patients who presented with hidradenitis suppurativa (HS) involving the axillary region and underwent surgical treatment between January 2017 and December 2024. Clinical, functional, and aesthetic outcomes were assessed in patients who underwent wide surgical excision of the entire hair-bearing area of the axilla followed by reconstruction with either a TDAP flap or a STSG.

Patients aged 17 years or older were eligible for inclusion if they had a diagnosis of Hurley stage II–III HS in the axillary region and had undergone reconstruction with either a TDAP flap or STSG following excision. Only patients with a minimum of six months of regular postoperative follow-up were included. Exclusion criteria were: secondary intention healing following excision, a history of systemic infection, immunodeficiency, or active malignancy, as well as incomplete medical records or insufficient follow-up data. Patients managed with primary closure or healing by secondary intention were excluded, as the present study specifically aimed to compare outcomes between flap-based and graft-based reconstruction following wide excision.

The diagnosis of axillary HS was established clinically based on typical lesion morphology, chronicity, recurrence, and axillary localization. Disease severity was classified according to the Hurley staging system during preoperative clinical examination. Hurley stage II was defined by recurrent abscesses with sinus tract formation and scarring, whereas Hurley stage III was defined by diffuse or near-diffuse involvement with multiple interconnected sinus tracts and abscesses. Histopathological examination of excised specimens was performed to support the diagnosis and exclude alternative pathology. Body mass index (BMI), smoking status, and comorbidities were recorded. This study was approved by the Ethics Committee of the Faculty (Approval no: 2025/740/12-24; date: 25 June 2025).

### 2.1. Surgical Technique

In all cases, wide excision was performed using a conventional scalpel and electrocautery; no laser-assisted excision was used. The excision encompassed all clinically visible sinus tracts and surrounding indurated tissue, irrespective of the planned reconstructive modality. For TDAP flap reconstruction, preoperative Doppler ultrasonography was used to identify perforators of the thoracodorsal artery. Once an appropriate perforator was identified, the pedicle was dissected, and the flap was harvested. Flap inset was performed only after completion of radical excision. The TDAP flap skin paddle was harvested from uninvolved tissue outside the resection field to minimize the risk of residual disease beneath the flap. The skin paddle was advanced in a V–Y fashion to cover the defect, and the donor site was closed primarily. The flap harvesting technique has been previously described in the literature [8,9].

In the STSG group, the defect was prepared, and a graft was harvested from the thigh. The graft was secured with tie-over dressing. Postoperatively, movement of the affected extremity was restricted for the first 48 h in both groups. Tie-over dressings were removed after 3 days in the STSG group, followed by daily wound care. Flap viability was assessed using color, capillary refill time, and temperature.

The reconstructive modality was not randomized. The choice between TDAP flap and STSG was individualized according to defect size, extent of axillary involvement, local tissue quality, wound-bed characteristics, patient comorbidities, perforator availability, and surgeon preference. TDAP flap reconstruction was preferred when durable pliable soft-tissue coverage was required and when suitable thoracodorsal artery perforators were identified preoperatively. STSG reconstruction was selected for defects with an adequate vascularized wound bed, particularly when grafting was considered sufficient for coverage or when flap reconstruction was not preferred because of patient-related or operative factors.

### 2.2. Outcome Assessment

Early postoperative complications (within the first 30 days), including partial or total flap/graft loss, infection, hematoma, and seroma, were recorded. Late outcomes included axillary range of motion, scar contracture, recurrence or sinus tract formation, and dermatology-specific quality of life assessed using the Dermatology Life Quality Index (DLQI). The DLQI is a 10-item dermatology-specific quality-of-life questionnaire with a total score ranging from 0 to 30, where higher scores indicate greater impairment. Scores are commonly interpreted as follows: 0–1, no effect; 2–5, small effect; 6–10, moderate effect; 11–20, very large effect; and 21–30, extremely large effect on the patient’s life.

### 2.3. Data Collection and Statistical Analysis

Data were obtained from patient files, electronic hospital records, and outpatient clinic follow-up notes. Statistical analyses were performed using Jamovi software, version 2.3. Categorical variables were analyzed using the chi-square test or Fisher’s exact test, while continuous variables were analyzed using Student’s *t*-test or the Mann–Whitney U test, depending on data distribution. Multivariate logistic regression analysis was performed to adjust for potential confounding variables, including comorbidities, BMI, and smoking status. A *p*-value < 0.05 was considered statistically significant.

## 3. Results

### 3.1. Patient Characteristics

A total of 35 surgical procedures were evaluated, comprising 15 reconstructions with a TDAP flap (42.9%) and 20 with STSG (57.1%). The mean age was 33.27 ± 8.03 years in the TDAP group and 37.70 ± 9.83 years in the STSG group, with no statistically significant difference (*p* = 0.152). The mean body mass index (BMI) was 28.36 ± 4.10 kg/m^2^ in the TDAP group and 32.69 ± 6.88 kg/m^2^ in the STSG group; although higher in the latter, the difference did not reach statistical significance (*p* = 0.074). Baseline characteristics are summarized in Table 1 and include demographic variables as well as disease-related factors relevant to reconstructive decision-making. No statistically significant differences were observed between groups with respect to age, sex, BMI, smoking status, or comorbidity distribution.

In the STSG group, there were 19 males (95.0%) and 1 female (5.0%), compared with 13 males (86.7%) and 2 females (13.3%) in the TDAP group (*p* = 0.565). Smoking was reported by 14 patients (70.0%) in the STSG group and 8 patients (53.3%) in the TDAP group (*p* = 0.512). Regarding comorbidities, 9 patients (45.0%) in the STSG group had none, while others had combinations such as hypertension (HT) + diabetes mellitus (DM) + chronic renal failure (10.0%), HT + DM + cardiomyopathy (10.0%), or HT + obesity (10.0%). In the TDAP group, 11 patients (73.3%) had no comorbidities, and the remaining comorbidities were less frequent. There was no significant difference between groups in comorbidity distribution (Table 1).

### 3.2. Surgical Details

The mean defect size was 105.47 ± 26.29 cm^2^ in the TDAP group and 164.65 ± 77.99 cm^2^ in the STSG group. Although larger defects were observed in the STSG group, the difference was not statistically significant (*p* = 0.116). The tendency toward larger defects in the STSG group likely reflects pragmatic reconstructive selection, with grafting preferred for more extensive defects, whereas TDAP flaps were used in defects amenable to perforator-based regional coverage.

The mean size of TDAP flaps harvested was 158.53 ± 41.44 cm^2^. No significant differences were observed between the two groups in terms of affected side (right/left/bilateral), use of vacuum-assisted closure (VAC), or distribution of defect sites (all *p* > 0.05). The mean follow-up duration was significantly longer in the TDAP group than in the STSG group (28.53 ± 16.38 months vs. 19.65 ± 28.06 months; *p* = 0.014). The follow-up range was 6–48 months in the TDAP group and 6–96 months in the STSG group, with median follow-up durations of 36 months and 6 months, respectively.

### 3.3. Quality-of-Life Outcomes

According to the post hoc ANOVA tests, both the TDAP and graft groups demonstrated significant improvements from preoperative to postoperative assessments (TDAP: mean difference, 20.87; 95% CI, 15.95–25.78; *p* < 0.0001; Graft: mean difference, 18.50; 95% CI, 14.25–22.75; *p* < 0.0001). No significant difference was observed between the groups in preoperative scores (*p* = 0.1687), and postoperative scores were likewise comparable, showing no statistically significant difference (*p* = 0.9608). These findings indicate that both techniques resulted in marked within-group improvements in quality-of-life scores, with no statistically significant between-group differences postoperatively.

Similarly, smokers showed a significant improvement in DLQI scores from preoperative to postoperative evaluations (mean difference, 20.73; 95% CI, 16.90–24.55; *p* < 0.0001). Preoperatively, smokers had significantly higher DLQI scores than non-smokers (mean difference, 5.72; 95% CI, 1.28–10.16; *p* = 0.0051), and their scores were also significantly higher than the preoperative values of non-smokers when compared postoperatively (mean difference, 23.18; 95% CI, 18.74–27.62; *p* < 0.0001). However, postoperative scores did not differ significantly between smokers and non-smokers (mean difference, 2.46; 95% CI, −1.98–6.89; *p* = 0.5908). Within the non-smoker group, a significant improvement from preoperative to postoperative measurements was also observed (mean difference, 17.46; 95% CI, 12.49–22.44; *p* < 0.0001). Collectively, these results suggest that although smokers exhibited greater preoperative impairment in quality of life, both smokers and non-smokers achieved substantial and comparable postoperative improvements.

### 3.4. Complications

Early postoperative complications (within 30 days) included flap or graft loss, infection, hematoma, and seroma. No partial or total flap loss, venous congestion, or seroma was observed, and flap survival was 100%. No hematomas were reported in either group. In the STSG group, graft loss occurred in 15% of cases, while in the TDAP group, wound dehiscence was observed in 13.3% of cases.

**Figure 1:**
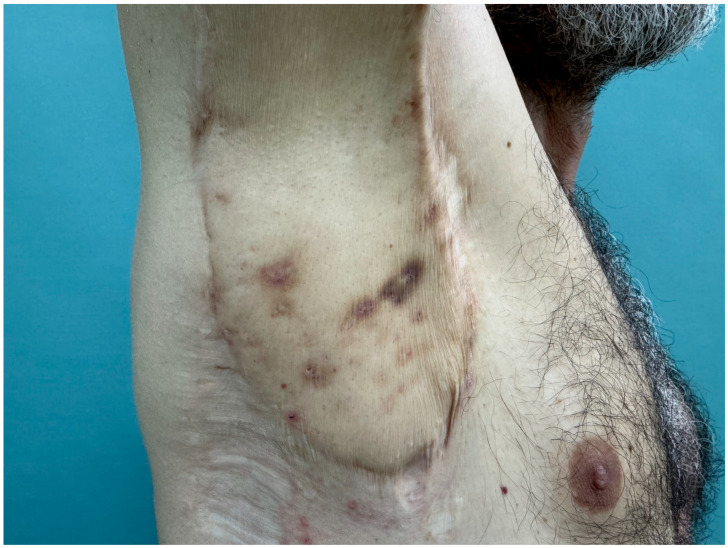
Long-term postoperative outcome after thoracodorsal artery perforator (TDAP) flap reconstruction for axillary hidradenitis suppurativa. Forty-eight-month postoperative view of a patient reconstructed with a TDAP flap following wide excision of axillary hidradenitis suppurativa. Stable soft-tissue coverage and preserved axillary contour are observed; however, local recurrence developed during long-term follow-up.

**Figure 2:**
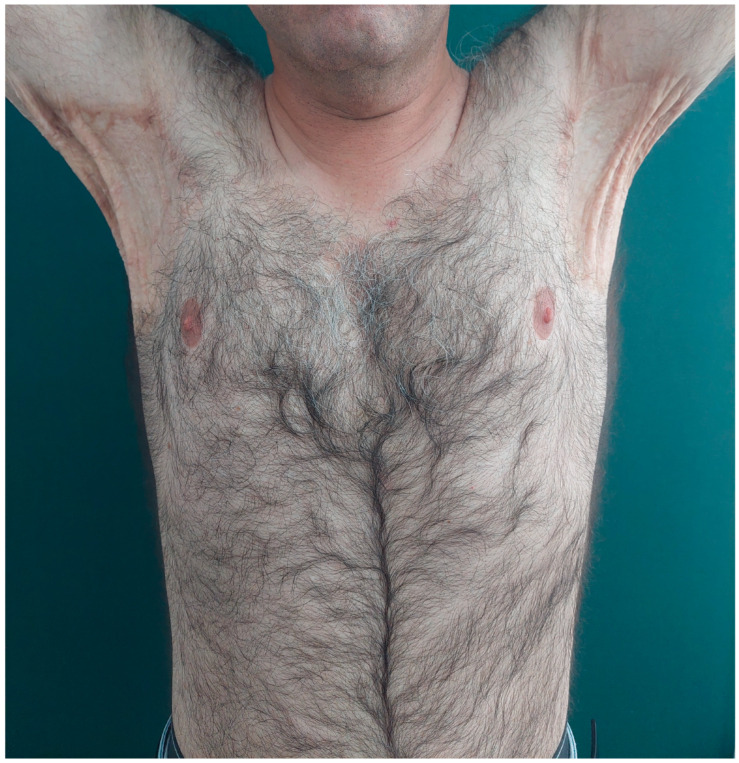
Long-term postoperative outcome after split-thickness skin grafting (STSG) reconstruction for axillary hidradenitis suppurativa. Ninety-six-month postoperative view of a patient reconstructed with STSG following wide excision of axillary hidradenitis suppurativa. The image demonstrates stable graft coverage of the axillary region without clinically evident recurrence during long-term follow-up.

**Figure 3:**
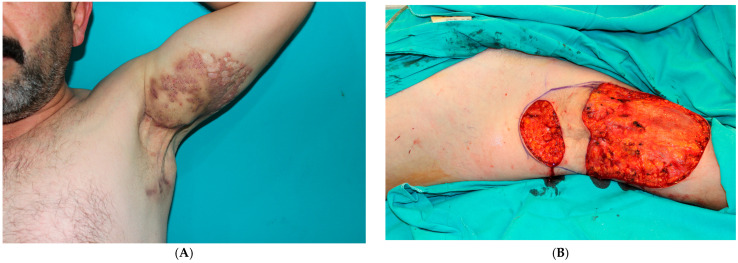

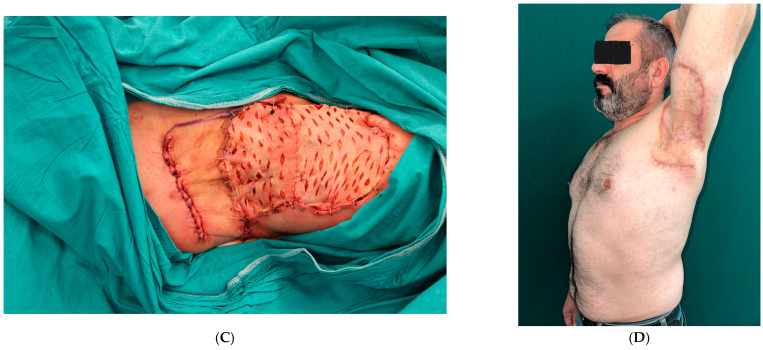
Clinical sequence of split-thickness skin grafting (STSG) reconstruction for axillary hidradenitis suppurativa. (**A**) Intraoperative view after wide excision of the diseased axillary tissue, demonstrating the extent of the resulting defect. (**B**) Prepared recipient wound bed before graft placement, showing adequate vascularized tissue suitable for split-thickness skin grafting. (**C**) Early postoperative appearance after STSG application, demonstrating successful graft take in the axillary defect. (**D**) Eighteen-month postoperative view showing stable graft coverage of the axillary region, with maintained wound closure and acceptable contour.

**Figure 4:**
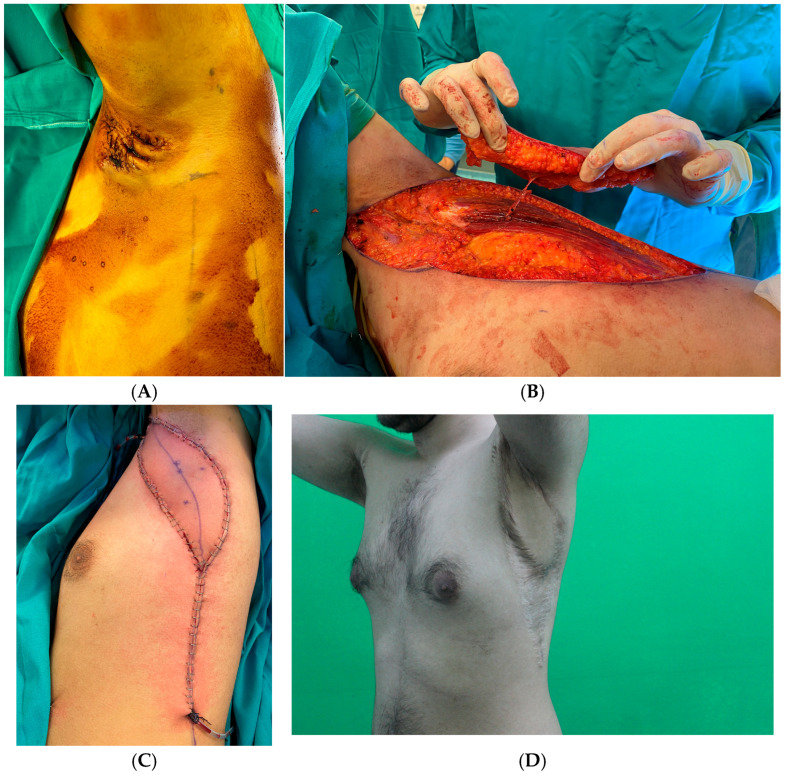
Clinical sequence of thoracodorsal artery perforator (TDAP) flap reconstruction for axillary hidradenitis suppurativa. (**A**) Preoperative view demonstrating axillary hidradenitis suppurativa involving the hair-bearing axillary region. (**B**) Intraoperative view after wide excision of the diseased tissue, including clinically visible sinus tracts and surrounding indurated tissue, showing the resulting axillary soft-tissue defect. (**C**) Intraoperative view following TDAP flap elevation and advancement, demonstrating flap inset for coverage of the axillary defect with well-vascularized regional tissue. (**D**) Twenty-four-month postoperative view showing stable soft-tissue coverage, preserved axillary contour, and satisfactory wound healing after TDAP flap reconstruction.

Late complications included assessment of axillary joint range of motion, scar contracture, recurrence, and sinus formation. Due to incomplete preoperative range-of-motion data, these measurements were excluded from statistical analysis. Postoperatively, shoulder abduction was ≥160° in all patients, with both groups demonstrating comfortable, functional motion and high satisfaction. Preoperative range-of-motion measurements were not uniformly available due to the retrospective nature of the study. Postoperatively, all patients demonstrated preserved shoulder mobility, with abduction ≥160° and no clinically significant restriction. No recurrences were detected in the STSG group, whereas 1 patient (6.7%) in the TDAP group had a recurrence (Figure 1, Figure 2, Figure 3 and Figure 4). None of the complication rates differed significantly between the two groups (all *p* > 0.05) (Table 2). Recurrence was observed in one patient in the TDAP group. This finding should be interpreted cautiously, as the TDAP cohort had a significantly longer follow-up duration, and the small sample size amplifies the impact of single events on percentage-based comparisons.

## 4. Discussion

In the present series, both TDAP flap and STSG reconstruction following wide excision provided durable wound coverage, low complication rates, and marked improvement in dermatology-specific quality of life. Although no statistically significant between-group difference was detected in postoperative DLQI scores or complication rates, these findings should not be interpreted as evidence of equivalence because of the retrospective design, limited sample size, and non-randomized selection of reconstructive technique.

The importance of adequate excision has been emphasized in previous studies. Soldin et al. reported lower recurrence after wider excision compared with hair-bearing area excision alone, supporting the concept that disease clearance and reconstructive planning should be considered together rather than as separate steps [10].

Healing by secondary intention has also been used after wide excision and avoids donor-site morbidity; however, it is associated with prolonged wound care, delayed return to daily activities, and the need for intensive follow-up or physiotherapy. Although acceptable scar quality and functional outcomes have been reported, recurrence and motion restriction remain concerns in some series [11,12]. For these reasons, reconstructive methods such as STSG and regional perforator flaps remain relevant options for axillary defects.

STSG remains a practical option for axillary reconstruction because it is technically straightforward, cost-effective, and suitable for large defects. Previous studies have reported favorable outcomes with both staged VAC-assisted grafting and single-stage STSG approaches [13,14]. In our cohort, STSG was commonly selected for larger defects with an adequate vascularized wound bed. However, graft-based reconstruction may require longer dressing care and may be more vulnerable to shear, moisture, delayed epithelialization, hypertrophic scarring, and secondary contracture in the axillary region.

Regional flap reconstruction has been advocated to provide durable, pliable, well-vascularized tissue in the axilla. Inner-arm and posterior arm flap modifications have been described with favorable functional and aesthetic outcomes [15,16,17,18]. Comparative studies have suggested that flap reconstruction may reduce healing time, recurrence, and shoulder restriction compared with graft-based approaches, although this may be offset by longer operative time and greater technical complexity [19].

Since its first description by Angrigiani et al. [20], the TDAP flap has been widely applied in both free and regional reconstructions. Most authors prefer meticulous perforator dissection to achieve adequate pedicle length, facilitating flap inset [8,9,21,22]. When a suitable perforator is lacking, a small segment of muscle may be included to ensure vascular reliability [23].

Previous studies have reported favorable functional and quality-of-life outcomes after TDAP flap reconstruction for axillary HS. Wormald et al. observed better quality-of-life scores with TDAP flap reconstruction compared with STSG, whereas Busnardo et al. reported improved shoulder abduction after TDAP flap reconstruction, although quality-of-life indices were not assessed [7,24]. Elgohary et al. also demonstrated reliable functional and aesthetic outcomes, with donor-site morbidity reported in approximately 10% of cases [25]. Our findings are consistent with these reports, showing preserved shoulder mobility and substantial postoperative improvement in DLQI after TDAP reconstruction.

Recent reviews also support the role of flap-based reconstruction in advanced axillary HS. Amendola et al. reported higher complication rates for skin grafts and lower rates for regional axial flaps, whereas Vaillant et al. found low recurrence and acceptable complication rates after perforator flap reconstruction [26,27]. In our series, complication rates were low in both groups, and no flap loss was observed. Nevertheless, the small sample size and unequal follow-up duration limit direct comparison with larger pooled analyses.

In our series, both TDAP flap and STSG reconstruction yielded favorable postoperative outcomes within the context of a non-randomized reconstructive strategy. The STSG group included larger defects on average, whereas the TDAP group had a significantly longer follow-up duration; therefore, direct comparison of late events, particularly recurrence, should be interpreted cautiously. The low complication rates observed in both groups and the absence of flap loss suggest that both techniques can provide reliable coverage when selected according to defect characteristics, wound-bed quality, and patient-related factors. The single recurrence observed in the TDAP group should not be interpreted as inferiority of flap reconstruction, as the small cohort size and longer follow-up in this group may have increased the likelihood of detecting late recurrence.

Beyond conventional surgical endpoints, the present study incorporated dermatology-specific quality-of-life assessment using preoperative and postoperative DLQI scores. Both groups demonstrated marked postoperative improvement, and mean postoperative DLQI scores corresponded to only a small residual effect on daily life. Postoperative shoulder abduction was preserved in all patients, indicating clinically acceptable functional status after reconstruction. Nevertheless, because preoperative range-of-motion data were incomplete, functional outcomes could not be analyzed as paired quantitative measures. This combined clinical and patient-reported evaluation may better reflect the multidimensional goals of axillary HS reconstruction, including durable coverage, disease control, preserved mobility, and improved daily life function.

From a practical standpoint, the choice of reconstruction should be individualized. STSG may be preferable for extensive defects, patients in whom a shorter or less technically demanding procedure is desired, or settings with limited resources. TDAP flap reconstruction may be advantageous when durable, pliable, well-vascularized tissue is required, particularly when shoulder mobility, scar quality, and long-term soft-tissue stability are major priorities. Because the present study was not randomized, these considerations should be interpreted as practical guidance rather than evidence of superiority of one technique over the other.

An important practical consideration in choosing a reconstructive strategy is postoperative wound care and overall healthcare expenditure. STSG typically requires prolonged postoperative dressing changes, close outpatient follow-up, and meticulous local wound care until complete epithelialization is achieved. This extended wound-care period may increase patient burden and delay return to daily activities. Nevertheless, from a healthcare system perspective, STSG remains a cost-effective reconstructive option, as it avoids longer operative time, microsurgical dissection, and potential donor-site morbidity associated with perforator flap surgery. Therefore, despite the need for prolonged dressings, STSG may represent a financially advantageous approach in selected patients, particularly in large defects or in settings with limited surgical resources, provided that adequate wound care and patient compliance can be ensured.

This study has several limitations. Its retrospective, single-center design limits generalizability. Reconstructive modality was not randomized and was determined by defect characteristics, wound-bed quality, perforator availability, patient-related factors, and surgeon preference, introducing potential selection bias. The sample size was relatively small, limiting statistical power; therefore, nonsignificant between-group differences should not be interpreted as equivalence between techniques. Follow-up duration was unequal between groups, which may have influenced recurrence detection. In addition, although the DLQI provided patient-reported information regarding dermatology-specific quality of life, a dedicated patient satisfaction scale was not used. Therefore, subjective satisfaction with scar appearance, axillary contour, donor-site morbidity, and overall reconstructive outcome could not be quantitatively assessed. Finally, incomplete documentation precluded detailed analysis of hospital stay and preoperative range-of-motion measurements.

## 5. Conclusions

In conclusion, both TDAP flap and STSG reconstruction after wide excision provided durable wound coverage and substantial improvement in dermatology-specific quality of life in patients with advanced axillary HS. No statistically significant difference was detected between techniques; however, this should be interpreted cautiously because of the limited sample size, retrospective design, and non-randomized treatment selection. Reconstructive choice should therefore be individualized according to defect characteristics, wound-bed quality, patient factors, perforator availability, and institutional resources.

## Figures and Tables

**Table 1:** Baseline demographic and clinical characteristics of patients undergoing TDAP flap or STSG reconstruction after wide excision for axillary hidradenitis suppurativa.

Variables	TDAP (n = 15)	Graft (n = 20)
Age (years)	33.27 ± 8.03	37.70 ± 9.83
BMI (kg/m^2^)	28.36 ± 4.10	32.69 ± 6.88
Gender		
Male	13 (86.7%)	19 (95.0%)
Female	2 (13.3%)	1 (5.0%)
Smoking		
Yes	8 (53.3%)	14 (70.0%)
No	7 (46.7%)	6 (30.0%)
Comorbidity: None	11 (73.3%)	9 (45.0%)

**Table 2:** Surgical characteristics, quality-of-life outcomes, and complications in the TDAP flap and STSG groups.

Variables	TDAP (n = 15)	Graft (n = 20)	*p*-Value
Defect size (cm^2^)	105.47 ± 26.29	164.65 ± 77.99	0.116
VAC use:			
YES	2 (13.3%)	4 (20%)	>0.05
NO	13 (86.7%)	16 (80%)	>0.05
Follow-up (months)	28.53 ± 16.38	19.65 ± 28.06	0.014
Preop DLQI	24.20 ± 2.37	20.45 ± 7.64	0.501
Postop DLQI	3.33 ± 4.51	1.95 ± 2.82	0.226
DLQI change	20.87 ± 3.74	18.50 ± 7.16	0.614
Early complication:			
–Any	2 (13.3%)	3 (15.0%)	0.085
–Graft loss	-	3 (15.0%)	
–Detachment	2 (13.3%)	0 (0.0%)	
Late complication: (Recurrence, Hypertrophic scar)	1 (6.7%)	0 (0.0%)	0.241

## Data Availability

The data presented in this study are available on request from the corresponding author.
